# Mortality from pulmonary hypertension in Europe 2001-2019

**DOI:** 10.1186/s12890-024-03235-y

**Published:** 2024-08-28

**Authors:** Adam Hartley, Harpreet Singh, Chinmay Jani, Justin D. Salciccioli, Joseph Shalhoub, Luke S. Howard, Dominic C. Marshall

**Affiliations:** 1 National Heart and Lung Institute, Imperial College, Hammersmith Hospital, London, UK; 2 Medical Data Research Collaborative, London, UK; 3 Division of Pulmonary and Critical Care Medicine, Medical College of Wisconsin, Milwaukee, WI USA; 4 Department of Medicine, Mount Auburn Hospital/Beth Israel Lahey Health, Cambridge, MA USA; 5 Harvard Medical School, Boston, MA USA; 6 Division of Pulmonary and Critical Care, Brigham and Women’s Hospital, Boston, MA USA; 7 Imperial College Healthcare NHS Trust, London, UK; 8 Department of Surgery and Cancer, Imperial College London, London, UK

**Keywords:** Pulmonary hypertension, Pulmonary arterial hypertension, Mortality, Europe

## Abstract

**Background:**

The incidence of Pulmonary Hypertension (PH) and Pulmonary Arterial Hypertension (PAH) is believed to be on the rise and is associated with poor outcomes.

**Methods:**

We extracted age-standardized mortality rates (ASMRs) for decedents ≥ 18 years of age from the World Health Organization Mortality Database, using International Classification of Diseases 10th edition codes for PH and PAH, covering the period from 2001 to 2019. The UK and European Union countries with at least 1,000,000 inhabitants and at least 75% of available data points over the study period were included.

**Results:**

Between 2001 and 2019, in countries with available data, the median ASMR for PH increased by + 1.19 per 1,000,000 (+ 22.51%) in females and + 0.36 per 1,000,000 (+ 6.06%) in males. Out of 19 countries, 13 demonstrate an increase in female PH ASMR, and 12 reported an increase in male PH ASMR. In contrast, median PAH ASMR decreased by -0.29 per 1,000,000 (-28.74%) in females and remained relatively unchanged in males, with a minor increase of + 0.01 per 1,000,000 (+ 1.07%). Notably, there was significant inter-country heterogeneity, with countries such as Hungary, Romania, and Poland displaying results incongruous with the rest of Europe.

**Conclusions:**

While publicly available mortality statistics for PH may be unreliable, these data suggest an overall increase in mortality across Europe from 2001 to 2019. However, mortality from PAH has shown a decrease in females and a modest increase in males. This underscores the urgent need for robust and high-quality mortality reporting, including international registries, for both PH and PAH.

## Introduction

Pulmonary Hypertension (PH), a condition characterized by elevated blood pressure within the pulmonary vasculature, is an uncommon disease associated with a poor prognosis. The diagnosis has historically centred on an invasively assessed mean pulmonary artery pressure ≥ 25 mmHg at rest with the right heart catheterization [1], although the threshold for diagnosis decreased to > 20 mmHg in 2018 [2]. PH is classified according to the World Health Organization (WHO) clinical categories, with WHO Group 1 representing pulmonary arterial hypertension (PAH), and WHO Groups 2-5 representing PH due to other causes [2].

Evaluating the epidemiology of PH is challenging due to its relative rarity, multifaceted aetiology, the requirement for invasive haemodynamics for confirmatory diagnosis, and patients often presenting late in the disease course. One retrospective echocardiographic study reported PH in 326 per 100,000 population, of which approximately 5% was attributed to PAH [3]. Assessing the incidence and prevalence of PAH is even more challenging, primarily due to a lack of disease awareness and likely underestimation in registry-based data, as these data heavily rely on reference centers for patient management [4]. Estimates for PAH incidence and prevalence range between 2.4 to 7.6 cases per 1,000,000 population per year and 15 to 30 cases per 1,000,000 population reported [4–6]. It is believed that the prevalence of PH is increasing. A Canadian study reported an increase from 99.8 to 127.3 cases per 100,000 population from 1993 to 2012, which is likely attributed to a genuine increase in incidence as well as improved access to echocardiography for non-invasive detection [7].

The prognosis of PH varies significantly according to aetiology, the presence of comorbidities, WHO functional class, and other clinical parameters. In the UK, the current 5-year survival rate is 51% for Group 1 PH (PAH) and ranges from 22 to 68% for Groups 2-5 [8]. Despite advances in PH management, including novel drug classes for PAH and nationalized specialist centres for comprehensive care, recent studies have demonstrated increasing mortality rates in both sexes in the USA [9, 10]. However, there is limited contemporary global data on changes in the incidence, prevalence, or mortality of PH or PAH [1].

Expanding on this critical issue, in this study, we aimed to analyse trends in mortality from PH and PAH across Europe over the last two decades (2001-2019) to understand the impact of increasing PH incidence on mortality. This is especially pertinent given the more inclusive threshold for PH diagnosis, as well as new therapies for PAH, WHO Group 3 interstitial lung disease-related PH, and WHO Group 4 chronic thromboembolic PH [11–13]. We assessed changes in mortality rates over time and evaluated any geographic or sex disparities that may have significant population health and economic implications for policymaking and healthcare resource planning.

## Methods

### Data sources

We extracted data from the WHO Mortality Database for the years 2001-2019 (https://www.who.int/data/data-collection-tools/who-mortality-database). The WHO mortality database is a publicly available resource compiled by the WHO from mortality data reported annually by member states from their local death registration systems. Data are reported as crude mortality, by cause, in each country per year. The WHO monitors data quality to ensure reliability and usability and provides a grading system on the quality of the data. For entry to the database, birth and population recording must exceed 90%. Full details of data collection and quality control are described elsewhere.

Disease-specific mortality for decedents ≥ 18 years of age was identified using the International Classification of Diseases, version 10 (ICD-10). ICD-10 code I27 was used for the extraction of all PH patients, with subcategories of I27.0 (Primary PH, representing PAH), I27.2 (Other secondary PH), I27.8 (Other specified pulmonary heart diseases), and I27.9 (pulmonary heart disease, unspecified).

We selected all European Union countries and the United Kingdom for initial data extraction and review of data completeness. Countries with > 25% (5/19 data points) missing data were excluded from the analysis. Furthermore, due to the relatively low incidence of this condition, we excluded countries with fewer than one million citizens (Cyprus, Luxembourg, and Malta).

### Ethical approval

This is a retrospective analysis of aggregated population data, and therefore, ethical approval is not required.

### Data handling

Crude mortality rates were extracted and stratified by age and sex. Crude rates were then age-standardized using the WHO standard population [14] by 5-year age groups to generate age-standardized mortality rates (ASMRs). Age standardization allows comparison over time as population structure changes and between countries, which may have variable demographics with respect to age. ASMRs are reported as deaths per 1,000,000 population per year, as this is the standard value for epidemiological studies of PAH.

### Statistical analysis

ASMRs were compared between the start and the end of the observation period in terms of crude and percentage difference. The start and end values were taken as the mean value for a 5-year period, 2001-2005 vs. 2015-2019. No statistical testing was performed to compare start and end values, as any difference seen represents complete population data rather than a sample of the population and can therefore be considered a "true" difference. Figures were generated using R Studio (2021.09.0), with plots generated using ggplot and locally weighted scatter plot smoothing with a span set to 0.9.

## Results

After excluding countries with missing data there were 19 and 13 countries included in the final analysis for PH and PAH, respectively.

### Mortality from pulmonary hypertension

**Fig. 1:**
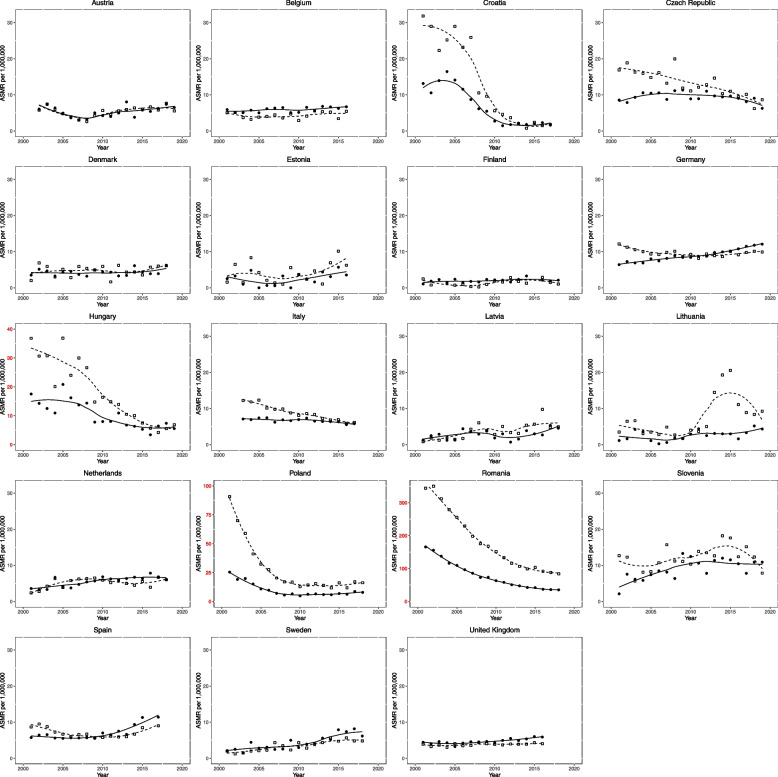
Trends in age-standardised mortality rates from pulmonary hypertension per million population 2001-2019 across Europe. (19 countries). Clear squares indicate males; filled circles indicate females. The lines (dotted for males, solid for females) represent locally weighted scatted plot smoothing with span set to 0.9. Axes are coloured in red to highlight differing scales compared to other plots

PH-related ASMR for males and females at the start and end of the observation period, along with changes over time, are presented in Table 1 and Fig. 1. The start is defined as 2001-2005, and the end is defined as 2015-2019 unless otherwise specified.

**Table 1:** Pulmonary hypertension age standardised mortality rate per million population and change over the observation period

Country	Start		End		Change	
	Male	Female	Male	Female	Male	Female
Austria	5.99	6.11	6.36	6.26	+ 0.36 (+ 6.06)	+ 0.15 (+ 2.53)
Belgium^a^	4.42	5.29	4.43	6.48	+ 0.01 (+ 0.17)	+ 1.19 (+ 22.51)
Croatia^b^	28.02	12.94	1.91	1.98	-26.11 (-93.17)	-10.96 (-84.73)
Czech Republic	16.73	9.06	9.09	8.43	-7.64 (-45.66)	-0.63 (-6.94)
Denmark^c^	4.98	4.42	5.36	4.62	+ 0.37 (+ 7.52)	+ 0.2 (+ 4.55)
Estonia^a^	3.42	1.70	8.20	4.62	+ 4.78 (+ 139.69)	+ 2.92 (+ 171.9)
Finland^c^	1.32	1.89	1.82	2.05	+ 0.5 (+ 38.36)	+ 0.16 (+ 8.45)
Germany	10.84	7.15	9.81	11.27	-1.02 (-9.43)	+ 4.12 (+ 57.64)
Hungary	33.77	16.29	5.96	5.62	-27.81 (-82.34)	-10.67 (-65.51)
Italy^b^	12.28	7.25	6.33	5.89	-5.95 (-48.48)	-1.36 (-18.69)
Latvia^c^	1.34	1.98	6.30	3.89	+ 4.96 (+ 371.68)	+ 1.91 (+ 96.24)
Lithuania	5.02	2.02	11.64	3.48	+ 6.62 (+ 131.9)	+ 1.46 (+ 72.58)
Netherlands^c^	3.33	3.57	5.54	6.77	+ 2.22 (+ 66.69)	+ 3.2 (+ 89.67)
Poland^c^	63.01	18.81	15.27	7.41	-47.74 (-75.76)	-11.4 (-60.61)
Romania^c^	315.29	142.16	91.80	37.63	-223.49 (-70.88)	-104.53 (-73.53)
Slovenia	9.73	5.80	13.05	10.31	+ 3.32 (+ 34.18)	+ 4.51 (+ 77.71)
Spain^b^	8.41	6.06	8.75	11.35	+ 0.34 (+ 4.09)	+ 5.29 (+ 87.33)
Sweden^c^	1.69	2.26	5.02	7.41	+ 3.33 (+ 197.34)	+ 5.15 (+ 228.15)
United Kingdom^a^	3.74	4.09	4.19	5.96	+ 0.45 (+ 12.13)	+ 1.87 (+ 45.6)

Start and end period are 2001-2005 and 2015-2019 unless otherwise specified. % change shown in brackets

^a^End data 2015-2016

^b^End data 2015-2017

^c^End data 2015-2018

At the end of the observation period, the median ASMR for PH was 6.26 per 1,000,000 for females and 6.33 per 1,000,000 for males, respectively. The countries with the highest mortality rates in females at the end of the observation period were Romania (37.63 per 1,000,000), Spain (11.35 per 1,000,000), and Germany (11.27 per 1,000,000). In males, the highest rates were observed in Romania (91.8 per 1,000,000), Poland (15.27 per 1,000,000), and Slovenia (13.05 per 1,000,000). The countries with the lowest PH ASMR in females were Croatia (1.98 per 1,000,000), Finland (2.05 per 1,000,000), and Lithuania (3.48 per 1,000,000). In males, the lowest PH ASMR was identified in Finland (1.82 per 1,000,000), Croatia (1.91 per 1,000,000), and the UK (4.19 per 1,000,000).

Over the observation period, the median change in PH ASMR was + 1.19 per 1,000,000 (+ 22.51%) in females and + 0.36 per 1,000,000 (+ 6.06%) in males. Out of the 19 countries, 13 demonstrated an increase in female PH ASMR, and 12 reported an increase in male PH ASMR. The countries with the greatest increases in ASMR for females were Spain (+ 5.29 per 1,000,000), Sweden (+ 5.15 per 1,000,000), and Slovenia (+ 4.51 per 1,000,000). For males, the greatest increases in ASMR were observed in Lithuania (+ 6.62 per 1,000,000), Latvia (+ 4.96 per 1,000,000), and Estonia (+ 4.78 per 1,000,000). Several countries saw a decrease in PH ASMR, with the most significant reduction in females occurring in Romania (-104.53 per 1,000,000), Poland (-11.40 per 1,000,000), and Croatia (-10.96 per 1,000,000). In males, the most substantial decreases over the observation period were seen in Romania (-223.49 per 1,000,000), Poland (-47.47 per 1,000,000), and Hungary (-27.81 per 1,000,000).

### Mortality from pulmonary arterial hypertension

**Fig. 2:**
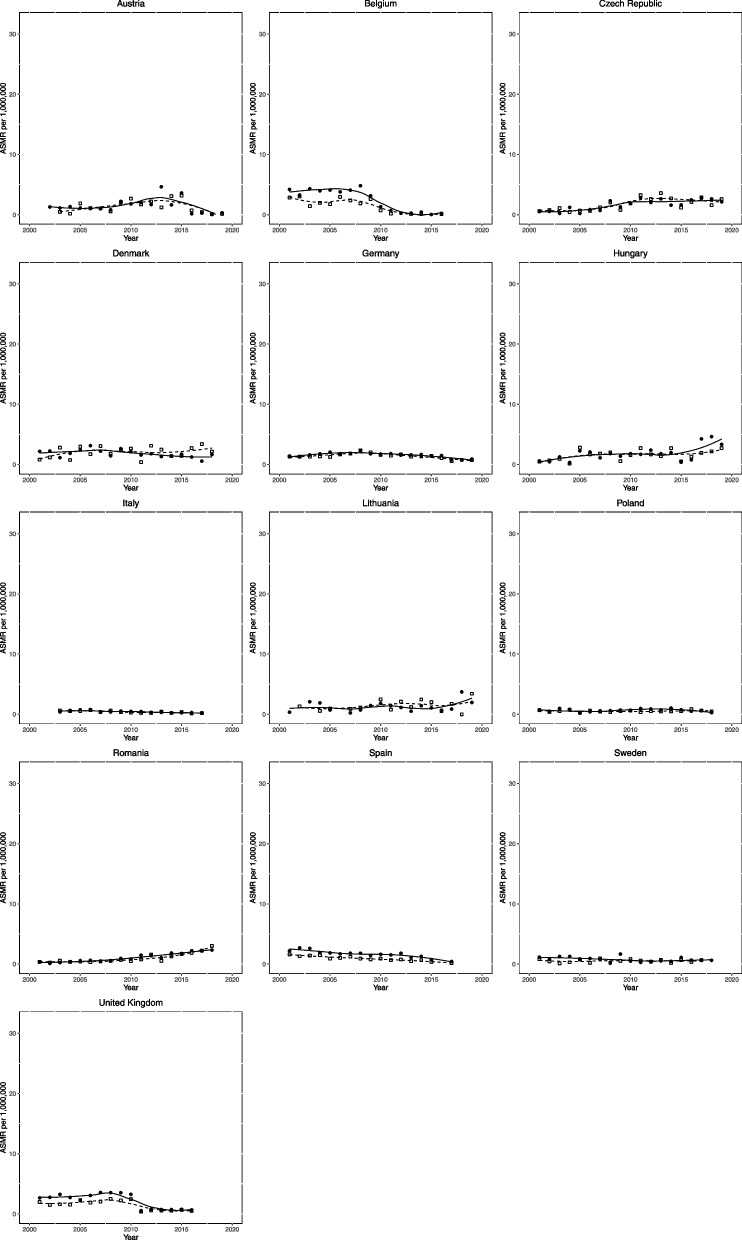
Trends in age-standardised mortality rates from pulmonary arterial hypertension per million population 2001-2019 across Europe. (13 countries). Clear squares indicate males; filled circles indicate females. The lines (dotted for males, solid for females) represent locally weighted scatted plot smoothing with span set to 0.9

PAH ASMR for males and females at the start and end of the observation period, along with changes over time, are presented in Table 2 and Fig. 2. The start is defined as 2001-2005, and the end is defined as 2015-2019 unless otherwise specified.

**Table 2:** Pulmonary arterial hypertension age standardised mortality rate (ASMR) per million population and change over the observation period

Country	Start		End		Change	
	Male	Female	Male	Female	Male	Female
Austria	1.17	1.17	0.90	0.87	-0.27 (-23.11)	-0.29 (-25.08)
Belgium^a^	2.27	3.97	0.10	0.13	-2.17 (-95.49)	-3.84 (-96.73)
Czech Republic	0.78	0.37	2.07	2.31	+ 1.29 (+ 165.11)	+ 1.94 (+ 521.73)
Denmark^b^	1.94	2.00	2.42	1.29	+ 0.48 (+ 24.71)	-0.71 (-35.38)
Germany	1.29	1.57	0.91	1.08	-0.38 (-29.65)	-0.50 (-31.51)
Hungary	1.23	1.14	1.73	2.68	+ 0.5 (+ 40.97)	+ 1.54 (+ 135.49)
Italy^c^	0.57	0.58	0.23	0.25	-0.34 (-59.61)	-0.33 (-56.68)
Lithuania	1.14	1.05	1.54	1.66	+ 0.4 (+ 35.42)	+ 0.61 (+ 58.45)
Poland^b^	0.48	0.58	0.63	0.53	+ 0.15 (+ 30.45)	-0.05 (-9.13)
Romania^b^	0.41	0.33	2.19	2.12	+ 1.78 (+ 431.84)	+ 1.8 (+ 546.82)
Spain^c^	1.31	2.34	0.27	0.59	-1.04 (-79.41)	-1.76(-75.04)
Sweden^b^	0.55	1.02	0.55	0.76	+ 0.01 (+ 1.07)	-0.26 (-25.74)
United Kingdom^a^	1.84	2.72	0.59	0.72	-1.26 (-68.21)	-2.00 (-73.44)

Start and end period are 2001-2005 and 2015-2019 unless otherwise specified. % change shown in brackets

^a^End data 2015-16

^b^End data 2015-2018

^c^End data 2015-2017

At the end of the observation period, the median ASMR for PAH was 0.87 per 1,000,000 for females and 0.90 per 1,000,000 for males, respectively. The countries with the highest rates of PAH ASMR in females at the end of the observation period were Hungary (2.68 per 1,000,000), the Czech Republic (2.31 per 1,000,000), and Romania (2.12 per 1,000,000). In males, the highest ASMR was reported in Denmark (2.42 per 1,000,000), Romania (2.19 per 1,000,000), and the Czech Republic (2.07 per 1,000,000). The countries with the lowest PAH ASMR for females were Belgium (0.13 per 1,000,000), Italy (0.25 per 1,000,000), and Poland (0.53 per 1,000,000). In males, the lowest PAH ASMR was reported in Belgium (0.10 per 1,000,000), Italy (0.23 per 1,000,000), and Spain (0.27 per 1,000,000).

Over the observation period, the median change in PAH ASMR was -0.29 per 1,000,000 (-28.74%) and + 0.01 per 1,000,000 (+ 1.07%) in females and males, respectively. Out of the 13 countries, 9 reported a decrease in PAH ASMR in females, and 6 reported a decrease in males.

In females, the countries with the greatest increases in PAH ASMR were the Czech Republic (+ 1.94 per 1,000,000), Romania (+ 1.8 per 1,000,000), and Hungary (+ 1.54 per 1,000,000). For males, the greatest observed increases in PAH ASMR were reported in Romania (+ 1.78 per 1,000,000), the Czech Republic (+ 1.29 per 1,000,000), and Hungary (+ 0.5 per 1,000,000). The majority of countries saw a decrease in females, with the greatest reductions in PAH ASMR reported in Belgium (-3.84 per 1,000,000), the UK (-2.00 per 1,000,000), and Spain (-1.76 per 1,000,000). The most substantial decreases in PH ASMR in males were seen in Belgium (-2.17 per 1,000,000), the UK (-1.26 per 1,000,000), and Spain (-1.04 per 1,000,000).

## Discussion

The aim of this study was to describe mortality from PH and PAH across Europe over the last 20 years. We identified an increase in PH mortality, particularly among females. Conversely, for PAH, mortality remained relatively unchanged for males, but there was an observed decrease in females.

One of the central messages derived from this study underscores the critical importance of data reporting, especially for relatively uncommon diseases such as PAH and PH. Such reporting is crucial for understanding changes in epidemiology and assessing the impact of new treatments or management approaches. Despite all the included countries having high-quality (except for Poland, which had medium-quality) cause of death data and a death reporting rate of over 99%, as assessed by the WHO [15], significant heterogeneity exists. Notably, Romania and Poland exhibit approximately 50- and tenfold increased ASMRs, respectively, compared to the European male median at the start of the observation period.

This substantial variation cannot be solely attributed to differences in disease prevalence, diagnostic availability, or therapeutic opportunities. Variations in coding practices may also play a role in these discrepancies, such as changes in ICD versions or ICD codes. For example, in 2003, the addition of ICD-10 code I27.2 (Other secondary PH) led to apparent significant decreases in PAH mortality in the USA [16]. Nonetheless, there is hope that as PH care continues to develop in national centralized specialist referral units [2], death reporting will become more standardized, thereby increasing data reliability. These centers should also facilitate the involvement of patients in national and international PH registries, providing essential "real-world" data for epidemiological and other research studies [17]. One such example is the multi-center "Comparative Prospective Registry of Newly Initiated Therapies for Pulmonary Hypertension" (COMPERA), which has now enrolled over 10,000 patients across 12 European countries [18].

The most recent ASMR for PH across Europe was 6.26 per 1,000,000 for females and 6.33 per 1,000,000 for males; whilst PAH was 0.87 per 1,000,000 for females and 0.90 per 1,000,000 for males, representing approximately 14% of PH ASMR for both sexes. Overall, the median ASMR for PH was observed to rise (22.51% in females and 6.06% in males) over the course of the study. These increases are particularly evident in Western European countries, including Germany, Spain, Sweden, and the UK. Potential explanations for these trends include the increasing incidence of PH, especially with the rising preponderance of WHO Group 2 (left heart disease) and WHO Group 3 (lung disease), which themselves have a poorer prognosis than other PH WHO Groups [7]. Thus, there is an emerging call for PAH-specific drugs to be trialed in patients in these categories, where those with left heart disease and chronic lung disease and coexistent PH experience significantly worse outcomes [19]. However, to date, only one drug, inhaled Treprostinil, has been approved in this space, specifically for PH associated with interstitial lung disease [12]. Many other studies have yielded negative results. The underlying reasons for the growth of these categories include population aging with accompanying comorbidities, such as obesity, obstructive sleep apnea, chronic obstructive pulmonary disease, hypertension, and ischemic heart disease. Additionally, increased awareness of the prognostic importance of PH in left heart and lung disease, coupled with the widespread use of Doppler echocardiography for non-invasive detection, has contributed to these observed increases in PH incidence and thus PH-related mortality. These trends are expected to expand further as diagnostic criteria become less stringent [11].

We also observed plateauing or declining mortality from PAH from 2001 to 2019 across Europe, with a median ASMR reduction of 28.74% in females and a very modest increase of 1.07% in males. This change is in line with the increasing life expectancy for PAH that has been documented in the UK [8, 20], but not observed in other WHO Groups. PAH prognosis is better than many other forms of PH, partially due to its younger age of onset and, perhaps more importantly, the availability of efficacious disease-modifying agents [21]. With newer therapeutic classes and combination therapies becoming increasingly established and initiated earlier in the disease course [17, 21], PAH mortality may continue to decline. However, there is a potential recent increase in PAH mortality in Eastern European countries that is not encountered in their Western counterparts, such as Hungary, Lithuania, and Romania, which may be due to less PAH-specific therapeutic availability in these regions. Interestingly, Hungary has few PH management centers listed with the European PH Association, while Romania is not included at all [22]. Sex disparities in mortality from PH (Fig. 1) and PAH (Fig. 2) are also readily observable. In Eastern European countries, male ASMR appears to be significantly greater than females, albeit decreasing over time as the sex gap narrows. Conversely, in Western European countries, no such trend is seen, with both sexes having similar mortality rates, or indeed, females having higher mortality rates. This would be expected, as PH, and certainly PAH, is more prevalent in females [23]. This is despite females often having an improved prognosis from PAH, termed the ‘oestrogen paradoxes’ [24]. However, PAH is increasingly diagnosed in older patients, where the traditional female preponderance is lost [25].

This study has several limitations, primarily stemming from its reliance on retrospectively collected population-level data, which can introduce bias due to reporting errors or misclassification of cause-of-death data, as discussed earlier. Additionally, as a purely descriptive observational study, we cannot infer causality from any of the data presented or related discussion. Furthermore, we lack the granularity of data needed to examine mortality across different aetiologies or mortality specifically related to PH caused by WHO Groups other than Group 1. Importantly, we are not commenting on case fatality rate, and thus trends in some instances may suggest increasing mortality rates, but that does not necessarily equate to reducing life expectancy in those with the diagnosis. Moreover, variations in healthcare system administration, levels of pulmonary hypertension care centralization, and coding/reporting methodologies across countries further complicate the interpretation of findings described here.

## Conclusion

Publicly available mortality statistics may not be entirely reliable for PH. Nevertheless, these data indicate a noteworthy trend across Europe from 2001 to 2019. Specifically, mortality rates from PAH have decreased in females and remained unchanged in males, while there are significant disparities in data from a few Eastern European countries that hinder comprehensive interpretation. This underscores the imperative need for robust and high-quality mortality reporting for both PH and PAH. This need is particularly crucial due to the currently underappreciated epidemiology of these conditions and the expected rise in incidence, driven by changes in diagnostic practices and the anticipated change in mortality associated with the advent of novel pharmacotherapies.

## Data Availability

All data reported here is freely available from the World Health Organisation, available here: https://www.who.int/data/data-collection-tools/who-mortality-database.
